# Training health providers to address unhealthy alcohol use in primary care: a cross-sectional, multicenter study

**DOI:** 10.1186/s12913-020-05730-4

**Published:** 2020-09-16

**Authors:** Esperanza Romero-Rodríguez, Luis Ángel Pérula de Torres, Roger Ruiz Moral, José Ángel Fernández García, Juan Manuel Parras Rejano, Ana Roldán Villalobos, Francisco Camarelles Guillem, Collaborative Group Alco-AP

**Affiliations:** 1 Maimonides Biomedical Research Institute of Cordoba (IMIBIC), Reina Sofia University Hospital, University of Cordoba, Córdoba, Spain; 2 Teaching Unit of Family and Community Medicine of Cordoba, Program of Preventive Activities and Health Promotion -PAPPS- (semFYC), Maimonides Biomedical Research Institute of Cordoba (IMIBIC), Reina Sofia University Hospital, University of Cordoba, Cordoba, Spain; 3 Villarrubia Health Center, Andalusian Health Service, Maimonides Biomedical Research Institute of Cordoba (IMIBIC), Reina Sofia University Hospital, University of Cordoba, Cordoba, Spain; 4 Villanueva del Rey Health Center, Andalusian Health Service, Maimonides Biomedical Research Institute of Cordoba (IMIBIC), Reina Sofia University Hospital, University of Cordoba, Cordoba, Spain; 5 Teaching Unit of Family and Community Medicine of Cordoba, Carlos Castilla Del Pino Health Center, Maimonides Biomedical Research Institute of Cordoba (IMIBIC), Reina Sofia University Hospital, University of Cordoba, Cordoba, Spain; 6 Infanta Mercedes Health Center, Madrid Health Service, PAPPS Health Education Group (semFYC), Madrid, Spain

**Keywords:** Training, Alcohol, Health system, Primary care, Health professionals

## Abstract

**Background:**

Health professionals’ training is a key element to address unhealthy alcohol use in Primary Care (PC). Education about alcohol use can be effective in improving PC provider’s knowledge and skills addressing alcohol-related problems. The aim of the study was to evaluate the training of health professionals to address unhealthy alcohol use in PC.

**Methods:**

An observational, descriptive, cross-sectional, multicenter study was performed. Location: PC centres of the Spanish National Health System (SNHS). Participants: Family physicians, residents and nurses completed an online questionnaire that inquired about their training (none, basic, medium or advanced), knowledge and preventive practices aimed at reducing unhealthy alcohol use. The study population was recruited via random sampling, stratified by the regions of the SNHS’s PC centre, and by email invitation to members of two Spanish scientific societies of Family Medicine.

**Results:**

A total of 1760 professionals participated in the study. Sixty-seven percent (95% CI: 67.5–71.8) reported not having received specific training to address unhealthy alcohol use, 30% (95% CI: 27.4–31.7) reported having received basic training, and 3% (95% CI: 2.3–4.0) medium/advanced training. The training received was greater in younger providers (*p* < 0.001) who participated in the PAPPS (Preventive Activities and Health Promotion Programme) (*p* < 0.001). Higher percentages of providers with intermediate or advanced training reported performing screening for unhealthy alcohol use (*p* < 0.001), clinical assessment of alcohol consumption (*p* < 0.001), counselling of patients to reduce their alcohol intake (*p* < 0.001) or to abstain, in the cases of pregnant women and drivers (*p* < 0.001).

**Conclusion:**

Our study reveals a low level of training among Spanish PC providers to address unhealthy alcohol use. A higher percentage of screening, clinical assessment and counselling interventions aimed at reducing unhealthy alcohol use was reported by health professionals with an intermediate or advanced level of training.

## Background

Alcohol use constitutes one of the leading preventable causes of morbidity and mortality [1] and represents one of the main preventable risk factors for non-communicable diseases, such as cardiovascular diseases, liver cirrhosis and various cancers [2]. Due to the health, social and economic impact of alcohol consumption, the World Health Organization aims to increase knowledge of the magnitude and determinants of alcohol-related harm and of interventions that help prevent and reduce alcohol’s impact on society [3].

Despite the effectiveness of alcohol training programs demonstrated in multiple studies [4–7], health professionals’ level of training on prevention and treatment of unhealthy alcohol use is low during their undergraduate and residency program, and in continuing education received throughout their clinical practice [8, 9]. This limitation in training constitutes one of the barriers to an alcohol prevention approach that health professionals face in their daily practice [10].

Recently, several international studies have been published evaluating the development of strategies to train health professionals to address the issue of alcohol consumption. Along this line, Anderson’s group [11] established the need to furnish Primary Care (PC) professionals with an adequate level of training and support to implement detection and intervention strategies for patients with unhealthy alcohol use. In addition, Keurhorst [12] reaffirms the concept of education/training as one of the basic keys to the acquisition of knowledge and preventive practices focused on screening and brief interventions in PC.

In Spain, several regional and national training strategies have been designed in the last decade aimed at improving approaches to alcohol among PC professionals, including the ARGOS Program [13] in Murcia, the Bebeu Menis training programme [14] in Catalonia, and the national programme “Mójate con el Alcohol” implemented by the Ministry of Health [15]. These training programs aim to inform health professionals about the importance of intervention at PC centres, and to increase their motivation and awareness of the problems arising from alcohol consumption, especially in adolescents, pregnant women and adults with unhealthy alcohol use. The programs favour the implementation of systematic interventions, through PC, for the prevention, and treatment of health problems related to alcohol use.

Several studies have shown the association between the levels of training held by health professionals on approaches to alcohol and their levels of preventive practice in the care they ultimately provide [16, 17]. Specifically, in the area of PC, training and support for health providers have a significant impact on the level of action on alcohol use subsequently attempted, as well as health professionals’ therapeutic commitment and attitudes towards reducing alcohol consumption in PC [18].

Several studies have addressed the training levels of health professionals from different health fields [19–21]. However, there are no published studies that address the level of training of Spanish PC professionals. Hence, a study is proposed with the following objectives: 1) To estimate PC professionals’ level of training on alcohol prevention, 2) To evaluate the relationship between the demographic and work-role related variables of the professionals and the levels of training they have.

## Methods

An observational, descriptive, cross-sectional, multicenter study was designed. The study’s population consisted of health professionals at PC centres across the Spanish National Health System (SNHS). The project was conducted from August 2014 to August 2016. The study was approved by the Ethics Committee of Cordoba’s Reina Sofía Hospital in 2014.

The selection criteria for the study were: to be a PC professional (family physician, nurse or family medicine resident) of the SNHS, and to agree to participate in the study.

The study population was recruited in three ways:

1. Emails to participants from a previous study, the ECAC (European Code Against Cancer) project [22], who had been recruited through PAPPS (Prevention and Health Promotion Activities Programme) and the Communication and Health Group, initiatives of the Spanish Society of Family and Community Medicine (semFYC).
2. Through the semFYC database and the Spanish Society of Primary Care Physicians (SEMERGEN), by sending emails to their members.
3. By means of a stratified random sampling of SNHS health centres, which was carried out based on the number of centres in each Autonomous Community. An email was sent to the director of the health centre, explaining the purpose of the study and encouraging him to publicise the study and have his team members complete the survey.

The list of centres was obtained from the Ministry of Health’s catalogue [23]. According to it, the number of health providers working in public PC was 33,482 and the number of health centres was 12,165. Assuming that 75% of the centres opted to collaborate on the study, with an average of 4 professionals per health centre, and 2 per local medical office, a sample of at least 430 local health centres and offices was deemed necessary.

The calculation of the sample size was made based on an alpha error of 5%, an accuracy of 3%, and an expected prevalence of training in alcohol prevention of 50% (p = q = 0.5; situation of maximum indetermination), it being necessary to include in the study at least 1068 professionals.

The survey was sent to 16,474 semFYC members and 8000 SEMERGEN members. Ultimately, 1110 members of semFYC and 469 members of SEMERGEN completed the questionnaire. The overall response rate, considering affiliation with scientific societies, was 6.4%.

The information was obtained through a questionnaire designed by members of Cordoba’s Family and Community Medicine Teaching Unit, with proven experience in the design and validation of surveys, and under the guidance of experts from the PAPPS (Preventive Activities and Health Promotion Programme, an initiative of the Spanish Society of Family and Community Medicine-semFYC-) [24]. The questionnaire developed for this study is provided as Additional File 1. This questionnaire was designed to be filled out independently and anonymously by each participating professional, after signing the informed consent form. It was subjected to a consensus-based process for the validation of its clarity, logic and content.

The study’s variables analysed were demographic (age; sex), professional (type of profession; resident trainer; time worked), training received on alcohol prevention (none, basic, medium or advanced), knowledge about alcohol consumption, and preventive practices aimed at reducing unhealthy alcohol use: identification of unhealthy alcohol use (screening for unhealthy alcohol use; clinical assessment), and counselling interventions (advice to reduce unhealthy alcohol use, counselling of pregnant women or drivers to abstain from alcohol).

The knowledge about alcohol consumption was measured by six self-reported items [concept of standard drink, concept of alcohol as a risk factor, concept of at-risk alcohol use (applied to men, and applied to women), concept of binge drinking (applied to men, and applied to women)]. Each item scored 0 (if the answer was incorrect) or 1 (if the answer was right). The overall knowledge of alcohol use was rated from 0 to 6. Preventive practices aimed at reducing unhealthy alcohol use encompassed detection of unhealthy alcohol use and counselling interventions. The identification of unhealthy alcohol use comprised two skills: clinical assessment and screening for unhealthy alcohol use in routine clinical practice; and counselling interventions encompassed advice to reduce unhealthy alcohol use and counselling of pregnant women or drivers to abstain from alcohol. Preventive practices aimed at reducing unhealthy alcohol use were reported according to the estimated percentage of patients for whom PC professionals provided clinical assessment, screening for unhealthy alcohol use, and counseling interventions. These practices were measured as follows: 0; 1–9%; 10–34%; 35–64%; 65–90%, 91–100%.

The surveys were completed online via Google Drive. The data was processed statistically with the SPSS (Statistical Package for the Social Sciences) v. 17.0 and EPIDAT 3.1 programs. A descriptive statistic was produced, and confidence intervals (95% CI) were calculated for the main estimators of the study. Subsequently, a bivariate analysis was carried out to verify the relationship between the independent variables and the questions on specific training to deal with alcohol consumption (Chi-square test, mean comparison test, such as Student’s t-distribution or ANOVA; after checking for normality –Kolmogorov-Smirnov test-, using bilateral contrasts, and a *p* ≤ 0.05).

A non-conditional multiple logistic regression analysis was performed to verify which variables were independently associated with the training received. The variables that were included in the regression model were: age, sex, type of professional, whether the professional is a participant in the PAPPS (these two treated as dummy variables), time worked in PC, and as resident trainers. Those variables whose *p* value with the Wald test was > 0.05 were eliminated, thereby obtaining the most parsimonious model. To verify the model’s goodness of fit, the Hosmer-Lemeshow test was used. Finally, a multiple linear regression analysis was carried out to ascertain which variables were associated with the level of knowledge regarding alcohol use, with age, sex, type of professional, time worked and being a resident supervisor being considered independent variables.

## Results

A total of 1760 PC professionals participated in the study, with a predominance of females (62.9%; 95% CI: 60.6–65.2). Of the respondents, 75.6% were family physicians, 12.5% nurses, and 11.4% family medicine residents. Their average age was 47.7 years [Standard Deviation (SD): 11.24; range: 26 to 64; 95% CI: 47.17–48.22), and their average time worked was 14.10 years (SD 10, 55; range: 1–39; 95% CI: 13.60–14.59). Twenty-six percent (95% CI 23.8–28.0) of health professionals were PAPPS members.

Sixty-seven percent (95% CI: 67.5–71.8) of respondents reported not having received specific training on alcohol prevention; 30% (95% CI: 27.4–31.7) said they had not received basic training on this subject, 2.6% (95% CI: 1.8–3.3) stated that they had received intermediate training, and 0.5% (95% CI: 0.02–0.09) reported advanced training. The evaluation of the levels of training depending on the sociodemographic and occupational variables, revealed lower percentages received training in those professionals over age 46 (*p* = 0.015), and in those who were not mentors (*p* = 0.003) (Table 1).

**Table 1:** Training on alcohol prevention among Primary Care professionals, according to sociodemographic and occupational characteristics

Sociodemographic and occupational characteristics of health professionals		Training on alcohol prevention				p*
		None n (%)	Basic n (%)	Medium n (%)	Advanced n (%)	
Age (years)	Younger than 35	290 (61.1)	168 (35.4)	16 (3.4)	1 (0.2)	0.015
	36–45	286 (66.2)	134 (31.0)	9 (2.1)	3 (0.7)	
	46–55	295 (69.2)	119 (27.9)	8 (1.9)	4 (0.9)	
	56 or older	307 (74.0)	94 (22.7)	13 (3.1)	1 (0.2)	
Sex	Female	734 (66.8)	332 (30.2)	29 (2.6)	4 (0.4)	0.566
	Male	444 (68.4)	183 (28.2)	17 (2.6)	5 (0.8)	
Type of provider	Family physician	888 (67.2)	391 (29.6)	35 (2.6)	8 (0.6)	0.242
	Resident	129 (62.0)	72 (34.6)	6 (2.9)	1 (0.5)	
	Nurse	161 (73.9)	52 (23.9)	5 (2.3)	0 (0)	
Resident trainer	No	777 (66.8)	350 (30.1)	35 (3.0)	2 (0.2)	0.003
	Yes	401 (68.7)	165 *(*28.3)	11 (1.9)	7 (1.2)	
Affiliated to PAPPS	No	760 (68.8)	313 (28.3)	27 (2.4)	5 (0.5)	0.004
	Yes	283 (62.5)	150 (33.1)	16 (3.5)	4 (0.9)	
	Do not know	135 (71.1)	52 (27.4)	3 (1.6)	0 (0.0)	

*The *p* values were obtained using the Chi-square test

The independent variables related to the PC professionals’ training in alcohol prevention, by means of logistic regression analysis, were: age (younger age of the professional, *p* < 0,001), type of provider (greater among family physicians and residents vs. nurses, *p* = 0.101) and being a PAPPS participant (*p* = 0.001) (Table 2).

**Table 2:** Variables associated with the level of training received regarding alcohol use. Logistic regression final model

Variables	B	p	OR	CI 95%
Age	−0.02	< 0.001	0.98	0.98–0.99
Type of provider		0.101		
-Family physician vs. nurse	0.35	0.036	1.42	1.02–1.97
-Resident vs. nurse	0.38	0.094	1.46	0.94–2.29
PAPPS affiliation		0.001		
-No vs. Do not know	0.28	0.120	1.32	0.93–1.89
-Yes vs. Do not know	0.66	0.001	1.93	1.30–2.85

Dependent variable: training on alcohol use; OR = Odds Ratio; 95% CI: 95% Confidence Interval; PAPPS = Preventive Activities and Health Promotion Programme; Hosmer and Lemeshow test = 8.485; *p* = 0.388

As shown in Table 3, health professionals with intermediate or advanced training on alcohol prevention reported higher levels of knowledge about alcohol use (ANOVA, *p* < 0.001).

**Table 3:** Knowledge of Primary healthcare professionals regarding alcohol use, according to the training received in this area

Training received	Average score	SD	CI 95%	Min	Max	p*
No training	2.49	1.82	2.39–2.59	0	6	< 0.001
Basic training	3.01	1.87	2.85–3.17	0	6	
Intermediate/Advanced Training	3.65	2.06	3.10–4.21	0	6	

*The *p* values were obtained via the ANOVA test

**SD: Standard Deviation; 95% CI = 95% Confidence Interval; Min = minimum; Max = maximum

The analysis of the PC professionals’ level of training in alcohol prevention and the identification of unhealthy alcohol use in PC revealed that those professionals with an intermediate or advanced level of training reported screening a higher percentage of their patients (p < 0.001) and providing clinical assessment for unhealthy alcohol use in a higher percentage (p < 0.001) (Table 4). Similarly, health providers who received intermediate or advanced training were more likely to advise their patients to reduce their alcohol consumption (p < 0.001), or to abstain from alcohol consumption, for pregnant women (p < 0.001) and for users of machinery or motor vehicles (p < 0.001) (Table 5).

**Table 4:** Training received on alcohol prevention amongst health professionals and their identification of unhealthy alcohol use in Primary Care

Identification of unhealthy alcohol use	Training received on alcohol prevention			
	None n (%)	Basic n (%)	Medium/Advanced n (%)	p*
Clinical assessment of unhealthy alcohol use**				< 0.001
0%	74 (6.3)	9 (1.7)	0 (0.0)	
1–9%	31 (26.3)	77 (15.0)	8 (14.5)	
10–34%	277 (23.5)	126 (24.5)	10 (18.2)	
35–64%	227 (19.3)	111 (21.6)	13 (23.6)	
65–90%	173 (14.7)	129 (25.0)	14 (25.5)	
91–100%	117 (9.9)	63 (12.2)	10 (18.2)	
Screening for unhealthy alcohol use**				< 0.001
0%	340 (28.9)	67 (13.0)	9 (16.4)	
1–9%	415 (35.2)	174 (33.8)	19 (34.5)	
10–34%	180 (15.3)	119 (23.1)	6 (10.9)	
35–64%	120 (10.2)	60 (11.7)	10 (18.2)	
65–90%	85 (7.2)	72 (14.0)	6 (10.9)	
91–100%	38 (3.2)	23 (4.5)	5 (9.1)	

*The *p* values were obtained using the Chi-square test

**Estimated percentage of patients for whom the primary care practitioner provided that activity

**Table 5:** Counselling interventions delivered by health professionals to reduce or abstain from alcohol use according to the training received on this subject

Counselling interventions to reduce/abstain from alcohol use	Training received on alcohol prevention			
	None n (%)	Basic n (%)	Medium/Advanced n (%)	p*
Counselling to reduce alcohol use**				< 0.001
0%	24 (2.0)	1 (0.2)	1 (0.2)	
1–9%	151 (12.8)	36 (7.0)	3 (5.5)	
10–34%	239 (20.3)	94 (18.3)	12 (21.8)	
35–64%	275 (23.3)	108 (21.0)	8 (14.5)	
65–90%	287 (24.4)	149 (28.9)	17 (30.9)	
91–100%	202 (17.7)	127 (24.7)	14 (25.5)	
Counselling pregnant women to abstain from alcohol use**				< 0.001
0%	57 (4.8)	7 (1.4)	0 (0.0)	
1–9	107 (9.1)	27 (5.2)	5 (9.1)	
10–34%	91 (7.7)	52 (10.1)	4 (7.3)	
35–64%	74 (6.3)	42 (8.2)	3 (5.5)	
65–90%	156 (13.2)	76 (13.2)	5 (9.1)	
91–100%	693 (58.8)	311 (60.4)	38 (69.1)	
Counselling operators of machinery, and motor vehicle drivers, to abstain from alcohol use**				< 0.001
0%	88 (7.5)	16 (3.1)	0 (0)	
1–9%	188 (16.0)	49 (9.5)	11 (20.0)	
10–34%	167 (14.2)	80 (15.5)	3 (5.5)	
35–64%	146 (12.4)	90 (17.5)	10 (18.2)	
65–90%	233 (19.8)	100 (19.4)	10 (18.2)	
91–100%	355 (30.2)	180 (35.0)	21 (38.2)	

*The *p* values were obtained using the Chi-square test

**Estimated percentage of patients for whom the primary care practitioner provided that activity

## Discussion

This study allows us to analyse Spanish health professionals’ levels of training on alcohol prevention in PC. We were also able to describe the association between training levels and awareness of this public health problem, and how this affects their preventive practices aimed at reducing alcohol consumption in PC. Our study reveals a low level of training among Spanish PC providers on approaches to address unhealthy alcohol use. In addition, health professionals with an intermediate or advanced level of training reported providing screening, clinical assessment and advice to reduce unhealthy alcohol use in a higher percentage of their patients.

PC health professionals are in an ideal position to address unhealthy alcohol use, as they are the most accessible in the health system, have a biopsychosocial viewpoint of patients and their families, and provide on-going care [25]. The current training program in the specialties of Family and Community Medicine and Nursing features a specific competence area for addressing problems related to substance use, including alcohol [26]. However, the continuing training that health professionals receive, once their residency period is over, is optional and not always focused on alcohol prevention in PC [27, 28].

Our study, concurring with a range of previous research, shows that the level of health professionals’ training focused on reducing alcohol consumption is low [29–32]. Of note are the findings indicating that the youngest professionals, and medical staff affiliated to PAPPS, benefit from a higher level of alcohol-related training. This could be due to the fact that during the period when doctors and nurses are resident interns, they do receive specific training on preventive approaches to alcohol consumption, but training in this area is scarce after finishing their residencies. These results highlight the need to bolster the continuing education of health professionals aimed at promoting health and preventing risk factors. Keurhorst et al. suggests the combination of multifaceted strategies to increase the delivery of alcohol screening and brief intervention and decrease patient’s alcohol use [33]. These multi-component strategies include organisational-, professional-, and patient-orientated interventions that can be used to implement the level of training on alcohol prevention among PC professionals (particularly in older physicians who have not received recent training on alcohol use).

According to a Delphi study [34] conducted on Spanish health professionals in 2007 on priorities in health promotion and prevention in PC, 84.3% of respondents stated that the consumption of alcohol, tobacco and illegal drugs is a preventive priority at PC facilities, followed by cardiovascular health problems (68.7%) and lifestyles (61.5%). The hurdles that PC health professionals face in preventive practice to tackle these health problems include a lack of training (59%), the attitudes of professionals towards prevention (18%) and the perception of limited usefulness of the interventions (18.1%). Taking into account the previous results, it follows that the training for professionals in the field of prevention and health promotion must take into account not only the transmission of knowledge about risk factors, but also increasing health professionals’ willingness to address this problem.

In relation to the healthcare profession, our study does not reveal a significant relationship between the training received on alcohol use and the different health professions, although our data does show that family physicians have higher levels of training on alcohol consumption than the nursing group. In 2018 *Wamsley* et al. [35] analysed the training differences between health professionals. In the case of doctors, the training received is oriented towards the diagnosis and treatment of acute and chronic diseases, with levels of postgraduate training on alcohol and other drugs varying depending on medical specialty. However, nursing training is focused on patient care, with a major practical component aimed at promoting health and preventing disease. This contrasts with our findings, since nursing reported less training in alcohol prevention than family physicians.

Studies have shown how PC professionals’ level of training has an impact on clinical practices aimed at reducing alcohol consumption [19, 36, 37]. Our findings reveal that health professionals’ training level affects the screening for unhealthy alcohol use, as well as their clinical assessment for this substance. Similarly, it is observed that professionals with more training are more likely to counsel their patients to reduce or abstain from alcohol use, particularly in cases of pregnant women and motor vehicle drivers. Given the importance of this finding in the clinical setting, strengthening the training of health professionals in this area should be a priority [38], in order to improve levels of prevention and health promotion, especially in at-risk populations, such as adolescents [39], pregnant women [40], and those whose alcohol consumption may have repercussions on third parties, such as operators of machinery and motor vehicle drivers [41].

### Strengths and limitations

This study has limitations that need to be considered. One of the difficulties encountered in measuring the health professionals’ level of training is the validity of the data obtained, as the degree of training was measured via voluntary declarations by the health professionals themselves, and not through more objective methods. This may have led to an overestimation of their levels of training. Another limitation of the study is the difficulty of comparing our data with that previously published on this subject, given the heterogeneity of criteria established to measure training received. This makes it difficult to establish consistent conclusions about health professionals’ training to deal with alcohol. In addition, it would be useful to know how frequently the participants received this training and identified patients with unhealthy alcohol use in the clinical setting. Furthermore, another limitation is the low participation rate identified in the present study. Our response rate of 6.4% contrasts with the overall participation of 27–54% published in European surveys [4, 42]. A possible explanation for this may lie in that professionals were uncertain about alcohol as an issue, due to the lack of training in alcohol prevention received during the residency program and the low continuing education received throughout their clinical practice.

Likewise, it is necessary to keep in mind possible selection bias, given the voluntary nature of the questionnaire, as the most motivated professionals may be more likely to answer it, which could distort the prevalence of training on the prevention of alcohol use [43]. Another limitation of the study may be the social desirability bias. It is possible that those who completed their training program may be less reluctant to admit they are not applying what they learned.

One of the strengths of our study, compared to others published in the field of PC, is its sample size. To date it is the largest work nationwide, and one of the largest at the international level. Furthermore, the present study represents one of the first nationwide analysis focused on the training of Spanish healthcare professionals in alcohol prevention, as well as their clinical practices aimed at reducing alcohol intake.

## Conclusions

Our study reveals a low level of training in alcohol prevention among Spanish PC professionals. For this reason, the need to develop preventive strategies, awareness-raising interventions, and training for health professionals on the risks of alcohol use and ways to reduce these risks in the general population, is evident. This fact is of great importance in the clinical field, given the significant influence that PC professionals have on the promotion of health and the prevention of unhealthy alcohol use.

## Supplementary information

**Additional file 1.**

## Data Availability

The datasets used/or analyzed during the current study are available from the corresponding author on reasonable request.

## Abbreviations

CI: Confidence Interval
ECAC: European Code Against Cancer
Min: Minimum
Max: Maximum
OR: Odds Ratio
PAPPS: Program of Preventive Activities and of Promotion of the Health
PC: Primary care
SD: Standard deviation
SEMERGEN: Spanish Society of Primary Care Physicians
semFYC: Spanish Society of Family and Community Medicine
SNHS: Spanish National Health System
SPSS: Statistical Package for the Social Sciences
