# Supplementary material for: Training health providers to address unhealthy alcohol use in primary care: a cross-sectional, multicenter study
Source: BMC Health Serv Res. 2020 Sep 16;20:877. doi: 10.1186/s12913-020-05730-4 (PMC7493933; doi:10.1186/s12913-020-05730-4)
Supplement: Supplementary file 1 — Additional file 1. [file 12913_2020_5730_MOESM1_ESM.docx]

Additional File 1.

**Knowledge about alcohol use**

**1.-What position do you think alcohol consumption occupies as a risk factor for premature death and disease, in relation to other factors such as tobacco, high blood pressure, and hyperlipidemia?**

- 1º Smoking, 2º Hyperlipidemia, 3º High blood pressure, 4º Alcohol
- 1º Alcohol, 2º High blood pressure, 3º Smoking, 4º Hyperlipidemia
- 1º Smoking, 2º High blood pressure, 3º Alcohol, 4º Hyperlipidemia
- 1º High blood pressure, 2º Alcohol, 3º Hyperlipidemia, 4º Smoking
- I do not know

**2.- “Standard Drink” is the average amount of alcohol ingested per unit of consumption, could you please tell me how many grams of alcohol a Standard drink contains (in Spain)?**

- 8 grams
- 10 grams
- 15 grams
- 17 grams
- I do not know

**3.- The risky alcohol use limit for men is:**

- 28 Standard drinks/per week
- 290 grams/per week
- 50 grams/per week
- 35 Standard drinks/per week
- I do not know

**4.- The risky alcohol use limit for women is:**

- 24 Standard drinks/per week
- 190 grams/ per week
- 35 grams/ per week
- 17 Standard drinks/per week
- I do not know

**5.- Definition of binge drinking (for men)**

- 55 grams/per day
- 6 Standard drinks/per week or more
- 100 grams/per day
- 12 Standard drinks/per day
- I do not know

**6.- Definition of binge drinking (for women)**

- 48 grams/per day
- 10 Standard drinks/per day
- 30 grams/per day
- 4 Standard drinks/per day
- I do not know

**TRAINING RECEIVED ON ALCOHOL PREVENTION**

**Have you received specific training in the last 5 years on alcohol prevention?**

o None

o Basic (for example, a course or workshop of 20-30 hours)

o Medium (for example, one course or workshop or several between 31 to 120 teaching hours)

o Advanced or expert (for example, courses with more than 120 teaching hours or a master's degree)

**PREVENTIVE PRACTICES AIMED AT REDUCING UNHEALTHY ALCOHOL USE**

**In your usual clinical practice, in what percentage, approximately, do you usually carry out these interventions with your patients?**

| **IDENTIFICATION OF UNHEALTHY ALCOHOL USE** | | | | | | |
| --- | --- | --- | --- | --- | --- | --- |
|  | 0% | 1-9% | 10-34% | 35-64% | 65-90% | >90% |
| Clinical assessment for unhealthy alcohol use |  |  |  |  |  |  |
| Screening for unhealthy alcohol use |  |  |  |  |  |  |
| **COUNSELLING INTERVENTIONS** | | | | | | |
|  | 0% | 1-9% | 10-34% | 35-64% | 65-90% | >90% |
| Counselling to reduce alcohol use |  |  |  |  |  |  |
| Counselling pregnant women to abstain from alcohol use |  |  |  |  |  |  |
| Counselling operators of machinery, and motor vehicle drivers, to abstain from alcohol use |  |  |  |  |  |  |

**SOCIO-DEMOGRAPHIC AND OCCUPATIONAL DATA**

**-Date of birth**

**-Sex**

- Male
- Female

**-Occupation**

- Physician
- Nurse
- Medical/Nursing resident
- Other

**-Resident trainer**

- Yes
- No

**-Indicate the years you have been working in Primary Care**

**-Region**

**-Do you belong to any scientific medical or nursing society?** Check "yes" or "no" for all answers

|  | Yes | No |
| --- | --- | --- |
| semFYC |  |  |
| SEMAP |  |  |
| SEMERGEN |  |  |
| SEMG |  |  |
| FAECAP |  |  |
| ASANEC |  |  |
| Other |  |  |

**-Are you affiliated to the PAPPS (Program for Preventive Activities and Health Promotion)?**

- Yes
- No
- I have not heard of PAPPS before
